# Ideas for how informaticians can get involved with COVID-19 research

**DOI:** 10.1186/s13040-020-00213-y

**Published:** 2020-05-12

**Authors:** Jason H. Moore, Ian Barnett, Mary Regina Boland, Yong Chen, George Demiris, Graciela Gonzalez-Hernandez, Daniel S. Herman, Blanca E. Himes, Rebecca A. Hubbard, Dokyoon Kim, Jeffrey S. Morris, Danielle L. Mowery, Marylyn D. Ritchie, Li Shen, Ryan Urbanowicz, John H. Holmes

**Affiliations:** 1 Department of Biostatistics, Epidemiology and Informatics, Perelman School of Medicine, University of Pennsylvania, Philadelphia, PA 19104-6116 USA; 2 Department of Genetics, Perelman School of Medicine, University of Pennsylvania, Philadelphia, PA 19104-6116 USA; 3 Department of Pathology and Laboratory Medicine, Perelman School of Medicine, University of Pennsylvania, Philadelphia, PA 19104-6116 USA

## Abstract

The coronavirus disease 2019 (COVID-19) pandemic has had a significant impact on population health and wellbeing. Biomedical informatics is central to COVID-19 research efforts and for the delivery of healthcare for COVID-19 patients. Critical to this effort is the participation of informaticians who typically work on other basic science or clinical problems. The goal of this editorial is to highlight some examples of COVID-19 research areas that could benefit from informatics expertise. Each research idea summarizes the COVID-19 application area, followed by an informatics methodology, approach, or technology that could make a contribution. It is our hope that this piece will motivate and make it easy for some informaticians to adopt COVID-19 research projects.

## Introduction

The coronavirus disease 2019 (COVID-19) pandemic has had a significant impact on population health and wellbeing. Research efforts are underway to identify vaccines [1], improve testing [2, 3], understand transmission [4], develop serologic tests [5], develop therapies [6], predict risk [7], and develop mitigation and prevention strategies [8, 9]. Biomedical informatics is central to each of these research efforts and for the delivery of healthcare for COVID-19 patients. Critical to this effort is the participation of informaticians who typically work on other basic science or clinical problems. The goal of this editorial is to highlight some examples of COVID-19 research areas that could benefit from informatics expertise. Each research idea summarizes the COVID-19 application area followed by an informatics methodology, approach, or technology that could make a contribution. This is followed by some practical suggestions for getting started. These are organized under sub-disciplines for biomedical informatics including Bioinformatics that focuses on basic science questions, Clinical Informatics that focuses on the delivery of healthcare, Clinical Research Informatics that focuses on research using clinical data, Consumer Health Informatics that focuses on the use of mobile devices and telemedicine, and Public Health informatics that focuses on research questions at the population or community level. It is our hope that this piece will provide motivation and make it easy for some informaticians to adopt COVID-19 research projects.

## Bioinformatics

We present here two applications of bioinformatics approaches to the basic science aspects of severe acute respiratory syndrome coronavirus 2 (SARS-CoV-2) and COVID-19. These focus on sequencing the virus, in order to understand the genomics of SARS-CoV-2 with the goal of informing treatment regimens and vaccine development.

### Genomic sequencing

The genome sequences of SARS-CoV-2 are essential to design and evaluate diagnostic tests, to track the spread of disease outbreak, and to ultimately discover potential intervention strategies. Phylogenetics is the study of the evolutionary connections and relationships among individuals or groups of species. These relationships can be identified through phylogenetic inference methods that evaluate the evolutionary origins of traits of interest, such as DNA sequences. Similar to tracing your ancestry through a DNA test, a phylogenetic analysis approach can be used to help map some of the original spread of the new coronavirus and trace a SARS-CoV-2 family tree based on its rapid mutations, which creates different viral lineages. Note that many countries have shared an increasing number of SARS-CoV-2 genome sequences and related clinical and epidemiological data via the Global Initiative on Sharing All Influenza Data or GISAID (https://www.gisaid.org). GISAID has generated a phylogenetic tree of 3123 SARS-CoV-2 genome samples between December 2019 and April 2020. In particular, Nextstrain, an open-source software package (https://nextstrain.org), uses SARS-CoV-2 genome data to help track the spread of disease outbreaks. For example, it could be applied to tell researchers where new cases of the coronavirus are coming from. This can be crucial information for investigating whether new cases arrived in given countries through international travel or local infection. One caveat is that the number of genetic differences among the SARS-CoV-2 genomes is close to the error rate of the sequencing process. Thus, there is a possibility that some of the observed genetic differences may be artifacts of this process. However, rapid data sharing for SARS-CoV-2 is the key to public health action and has led to faster-than-ever outbreak research. With more data sharing of the SARS-CoV-2 genomes, more genetic diversity will become apparent making it possible to better understand how the coronavirus is being transmitted.

### Genetics

While exploring the genome sequence of the SARS-CoV-2 virus is anticipated to provide scientists a better understanding of viral evolution and aid in the development of vaccines and treatments, evaluation of host genetics in response to COVID-19 is of similar importance. For other viruses, we know that some individuals have a natural immunity whereby even when exposed to the virus, they do not develop infection. For example, the well-known *CCR5-delta32* allele has a variation that protects individuals who have been exposed to the Human Immunodeficiency Virus (HIV); they are protected from developing AIDS (Acquired Immunodeficiency Syndrome) [10]. Because of this, researchers are gearing up to study the genomes of COVID-19 positive patients in comparison to controls (COVID-19-negative patients). For example, Stawiski et al. investigated coding variation in the gene, *ACE2*. ACE2, the human angiotensin-converting enzyme2, is a cell surface protein that the viral spike coat protein SARS-CoV-2 engages to invade the host cell [10]. What would be optimal for these and other genome-wide analyses, to identify potential risk and protective variants, are individuals who test positive for the virus but remain asymptomatic. These individuals will be more difficult to identify because of the lack of widespread testing (most individuals without symptoms are not being tested). However, the research community is building large, international, collaborative consortia to address this challenge, such as the COVID-19 Host Genetics Initiative (https://www.covid19hg.org/). Much like understanding the viral genome will be useful for drug development, identifying the genetic variation in the host DNA that is either increasing the risk of, or protection from, SARS-CoV-2 infection will enable us to identify putative targets for therapeutics and vaccines.

## Clinical informatics

We present here three topics relevant to the diagnosis and management of COVID-19 patients. These include imaging, suggestions for the roles that informaticians can assume in the pandemic, and the need for novel approaches to delivering patient care and learning from in-practice data.

### Imaging

Imaging provides a powerful tool for COVID-19 diagnosis and patient monitoring given the impact on lung physiology and anatomy. For example, chest computed tomography (CT) has been shown to have promising sensitivity and early detection power compared with the standard reverse transcriptase polymerase chain reaction (RT-PCR) test [11]. In addition, imaging plays an important role in assessing patients with worsening respiratory status [12], which is crucial for monitoring and treatment planning. Given the fast-growing volume of COVID-19 cases, to help alleviate the huge manual evaluation burden on clinicians, there is an urgent call for researchers in imaging informatics (or radiomics) to work on developing automated image analysis and artificial intelligence (AI) methods and tools. To achieve these goals, major efforts have been initiated to address two critical research foci. The first is to create large-scale high-quality imaging data repositories (e.g., Radiological Society of North America (RSNA) COVID-19 Imaging Data Repository, https://www.rsna.org/COVID-19) to accelerate collaborative research on image-based COVID-19 diagnosis and treatment. The second is to develop innovative AI methods for automatic image analysis for COVID-19 diagnosis and severity assessment. To get started on supporting these efforts, below we suggest a few relevant resources for interested imaging informaticians. Several COVID-19 resource and initiative web portals have been created by major organizations such as American College of Radiology (ACR), Radiological Society of North America (RSNA), and European Society of Medical Imaging Informatics (EuSoMII). These portals offer important information on policies, guidelines, discoveries, initiatives, data sets, and/or other relevant resources. Given the rapidly growing AI-based imaging literature on COVID-19, it is worth noting a recent review article [13], which provides comprehensive coverage on a variety of interesting topics, including AI-empowered contactless imaging workflow, AI in lung image segmentation, AI-assisted diagnosis and severity assessment, AI in follow-up studies, public imaging datasets for COVID-19, and future directions.

### Roles for informaticians in the pandemic and beyond

To effectively address the ever-growing surge of COVID-19 patient cases, informatics solutions are being developed to help care providers and healthcare institutions manage patients from symptoms to recovery. Symptom screening tools have been developed to aid patients in distinguishing COVID-19 symptoms from common colds and flu. Telemedicine is helping keep patients at home by deploying chatbots to answer patient COVID-19 questions and providing virtual visits and consultations to limit the number of individuals exposed to COVID-19 and to manage patients with mild COVID-19 symptoms. This reduces resource utilization and overburden on the care delivery system. Capacity and resource management tools can generate projects based on regional infection counts and current patient admissions to estimate the number of patients that will require hospitalization, intensive care unit beds, medications, and mechanical ventilation. These projections can improve clinical response times and inform triage care strategies. Donation and resource inventory tools can be helpful for identifying, cataloging, and distributing personal protective equipment (PPE), homemade masks, and other critical medical supplies to those fighting on the front lines. Informaticians can support these efforts by 1) educating patients and care providers about data science resources and electronic health record (EHR) platforms for building point-of-care solutions, 2) joining the open-source community efforts to develop these technologies, and 3) volunteering with the information services divisions within their healthcare organizations to deploy telehealth tools and engage in patient management projects.

### Clinical information systems and learning healthcare systems

The COVID-19 pandemic has been an unprecedented stress test for clinical information systems. The scramble to develop and implement new clinical practices has in many cases outpaced our ability to effectively use standard tools for building, testing, and monitoring these practices. For instance, clinical laboratories have rapidly implemented several different methods for SARS-CoV-2 diagnostic testing and have also needed to send out testing to multiple reference laboratories. These complex practices have made it non-trivial to collect even the most basic information, including who is being tested and who is positive. These data are essential both to the care of individual patients and to health providers who need to design these care systems and plan for what is coming next. These data are also being reported to government agencies in multiple new manual processes. The work that has been done to build these data collection systems is extraordinary and commendable. But, for our clinical informatics and public health communities, these challenges highlight the need for developing modern, flexible clinical information systems and robust infrastructure for inter-institution data sharing.

The implementation of novel clinical practices has also been notable for how much we still do not know about their clinical utility. As a consequence, there is a great need to learn about clinical utility from in-practice data. For example, the precise clinical sensitivity and clinical specificity of the SARS-CoV-2 diagnostic testing being used are currently unclear [14]. This is critically important because false-negative results could lead to the inappropriate non-use of PPE or insufficient clinical and epidemiological monitoring. The rate of such false-negatives is also highly variable across time, as the disease prevalence changes, and across multiple patient, provider, and geographic factors. To fill in these knowledge gaps, there is a big need for the design and application of methods for estimating such parameters from in-practice data. These approaches must be robust to the many sources of bias in these kinds of retrospective data and must be applied to datasets of large enough sample sizes, to generate meaningfully precise estimates.

## Clinical research informatics

We present here four clinical research informatics domains related to the generation, integration, and use of clinical and other data that could be leveraged in addressing the pandemic in various settings. The domains include a well-developed informatics infrastructure that encompasses a large healthcare landscape, the potential for systematically and cautiously repurposing drug treatments, the leveraging of existing clinical and biospecimen data, and the role of advanced statistical, integrative, and machine learning (ML) tools for diagnosis and treatment.

### Informatics infrastructure

One critical need to support COVID-19-related clinical and translational research studies is the development of informatics infrastructure that contains accurate and timely clinical data from the electronic health records of the COVID-19 population. As a first step, healthcare institutions can create patient registries to maintain reliable lists of COVID-19 patients and cases (e.g., confirmed, ruled out, uncertain). These data must be updated regularly (daily or several times each week) and contain a broad set of data elements representing demographics, prior medical histories, current medications, comorbidities, diagnoses, procedures, outcomes, etc. to serve a broad base of clinical investigators and scientific inquiries. To adequately code all patient data, image processing will be needed to encode salient radiological findings, and natural language processing will be needed to extract symptom onset, severity, and duration among other variables. Secure informatics platforms such as Integrating Bench to Bedside (i2b2) and the Shared Health Research Information Network (SHRINE), Trinetix, and Atlas play an important role in standardizing and harmonizing clinical data to common data models (CDMs) including i2b2, Patient-Centered Outcomes Research Network (PCORnet), Fast Healthcare Interoperability Resource (FHIR), and Observational Medical Outcomes Partnership (OMOP). Once COVID-19 patients are indexed within the patient registry and their clinical data has been extracted, transformed, and loaded into these frameworks, clinical researchers can execute secure, privacy-preserving, and federated queries across all participating sites using any framework to identify patients for clinical trials, generate scientific hypotheses, and conduct observational studies. Both aggregate and individual-level information can be made available with appropriate data governance, ethical review, and institutional agreements. Informaticians can support these efforts by 1) developing technologies and algorithms for extracting, encoding, and mapping raw EHR data to emerging COVID-19-specific CDMs, 2) engaging in existing and emerging consortiums, both grass roots and nationally-sponsored efforts, across Clinical and Translational Science Awards (CTSAs) and informatics networks, and 3) connecting with clinicians to develop and share informatics tools and predictive models that identify clinically-formative, actionable insights from heterogeneous, temporal data.

### Pharmacovigilance and repurposing existing data for COVID-19 treatments

One of the major challenges with emerging diseases, such as COVID-19, is that evidence for effective drugs and treatments is sparse. While vaccine development is important, vaccines are only helpful to prevent individuals from becoming infected in the first place. For those that have COVID-19, the main strategy for treatment with drugs (while the disease is still emerging) is to reuse those that have been approved for other purposes. There are several drugs that may therapeutic use in COVID-19, namely: hydroxychloroquine sulfate, chloroquine phosphate, remdesivir, carfilzomib, eravacycline, valrubicin, lopinavir and elbasvir. These medications were designed for treatment of various diseases, including lupus, malaria, cancer and HIV. Therefore, the use of these medications to treat COVID-19 is termed ‘drug repurposing’ and one avenue for studying the potential for a drug to be repurposed is through informatics.

Informatics methods have been developed for both drug repurposing and pharmacovigilance (studying the adverse effects of a drug). The advantage of using existing EHRs for studying drugs as candidates for drug repurposing is that it enables risk assessment profiles to be generated for each candidate drug. Since the drugs have been prescribed previously during routine clinical care, it is possible to study their effects on human health in a variety of situations that may not have been included in the original clinical trials. For example, the birth and pregnancy outcomes following drug exposure can be assessed using EHRs for drugs potentially useful in treating COVID-19, such as hydroxychloroquine. This is important as the hydroxychloroquine clinical trials for COVID-19 specifically exclude pregnant women from enrolling in their studies. Informatics methods can also be designed, which use more sophisticated machine learning and artificial intelligence methods to study the effects of medication exposure during pregnancy on fetal and maternal outcomes [15].

### Data integration

With the aggregation of clinical and medication data from EHRs, along with the recruitment of COVID-19 positives and negatives for genetic studies (as described above), there is an opportunity to explore genetic data in combination with this EHR data to improve our understanding of the COVID-19 disease’s severity and outcomes. Early research has suggested that individuals, who have medical conditions such as heart disease, diabetes, obesity, or asthma, may be at higher risk for severe disease and/or worse outcomes from COVID-19. Additionally, early data suggests that some medications such as ACE-inhibitors, angiotensin release blockers (ARBs), or non-aspirin nonsteroidal anti-inflammatory drugs (NSAIDS) may be linked to worse health outcomes due to COVID-19. However, these reports are primarily based on small, observational datasets without rigorous, epidemiological study designs. As such, these associations are met with much controversy in the literature. With the accumulation of COVID-19 positives and negatives, along with access to EHR data, including comorbid conditions and medications, researchers will be able to develop more thorough studies of which medical conditions are associated with poorer COVID-19 outcomes and/or which medical conditions place individuals at higher risk for hospitalization due to COVID-19. Additionally, if these data are paired with genetic data from EHR-linked biobanks, we may be able to determine if some of these differences in COVID-19 severity and/or outcomes related to comorbidities and medications are also related to host genetics. Fortunately, there are several efforts to establish data-sharing consortia that provide an opportunity for informaticians to assist with analyses. For example, the Consortium for Clinical Characterization of COVID-19 by EHR, or 4 CE (https://covidclinical.net/), has released summary-level COVID-19 data from several countries including France, Germany, Italy, Singapore, and the United States along with a preprint of the initial analyses [16].

### Advanced computational approaches to diagnosis, decision making, and treatment

Presently, there is much to be learned regarding how best to treat COVID-19 patients when sufficient resources are available, as well as how to optimize operational decisions such as the triage of patient testing and care when they are not. As accessible, cleaned, and structured EHR data become available for COVID-19 patients at both the institutional and multi-site consortium levels, there will be increased opportunity to apply machine learning to better understand and make risk predictions on a variety of clinically and operationally relevant outcomes. The accessibility of data science and ML packages (e.g. Pandas, scikit-learn, and TensorFlow Python libraries), paired with widely available high-powered computational hardware offers significant opportunity for researchers to get involved in data analysis and modeling. However, many caveats need to be taken into consideration in order to develop and apply effective, rigorous ML analysis pipelines for replicable COVID-19 investigation.

Some key considerations and targets of research include: (1) feature engineering, transforming raw data into features (i.e. variables) that ML can better utilize to represent the problem/target outcome, (2) feature selection, applying expert domain knowledge, statistical methods, and/or ML methods to remove ‘irrelevant’ features from consideration and improve downstream modeling, (3) data harmonization, allowing for the integration of data collected at different sites/institutions, (4) handling different outcomes and related challenges, e.g. binary classification, multi-class, quantitative phenotypes, class imbalance, temporal data, multi-labeled data, censored data, and the use of appropriate evaluation metrics, (5) ML algorithm selection for a given problem can be a challenge in itself, thus strategies to integrate the predictions of multiple machine learners as an ensemble are likely to be important, (6) ML modeling pipeline assembly, including critical considerations such as hyper-parameter optimization, accounting for overfitting, and clinical interpretability of trained models, and (7) considering and accounting for covariates as well as sources of bias in data collection, study design, and application of ML tools in order to avoid drawing conclusions based on spurious correlations.

Advanced tools may be necessary to deal with data analytic challenges, properly analyze these data, and accurately extract the knowledge embedded in them. Some key challenges include: (1) accounting for correlation structure induced by multi-level, spatial, and longitudinal designs, (2) adjusting for biases emanating from the observational data using causal approaches, (3) accounting for privacy-induced limitations on the resolution of data that can be shared, and (4) discovering and characterizing interpatient heterogeneities in incidence, progression, or response through stratified or latent class models. Some of these challenges can be handled by aptly chosen existing methods, while others require new methodological development. The COVID-19 crisis and the extensive data resources that it will produce will provide an excellent opportunity to develop such methods, including privacy-preserving integrative analytical tools as well as advanced causal inference tools that also account for these other data complexities.

## Consumer health informatics

We present here two related approaches to using informatics solutions, which directly involve the public who are not physically situated within a healthcare setting. The first focuses on using smartphones and other technology for educating the public about the pandemic and ways to avoid infection as well as monitoring, and the second explores the use of sensors in this domain.

### Patient education and monitoring

Consumer health informatics, focusing broadly on tools and systems that engage and empower patients and more general health consumers in health delivery and decision making processes, has a substantial role to play in the context of a pandemic. Specific areas that consumer informatics researchers and system designers can target include consumer education, self-triage, monitoring, and social engagement. In a time when behavioral guidelines are continuously adjusted based on new data, *consumer education* is essential to conveying and disseminating actionable and timely information. Patient portals and other web sites can provide educational content that can be tailored to individual information needs as well as literacy and health literacy levels. Furthermore, systems can include an interactive component that can facilitate decision support and *self-triage*. One such example is a patient portal for self-triage and scheduling that was created at the University of California San Francisco to enable asymptomatic patients to report exposure history and for symptomatic patients to be triaged and paired with appropriate levels of care [17]. The system is already being used extensively and performs with high sensitivity in recommending emergency-level care for symptomatic patients. It also prevents unnecessary visits. Tools that have been traditionally used for *patient monitoring* at home and the community can also be useful in generating data that provide insight into disease spread and health needs. An example is that of a smart thermometer vendor that has created an app which allows users to record their temperature and other symptoms with a Health Insurance Portability and Accountability Act (HIPAA) compliant platform; data are aggregated and demonstrate how the virus moves from one county to another, providing a detailed visualization map that highlights areas with an unusually high number of recorded prevalence of fever (https://healthweather.us/).

Other mobile health tools that track aspects of daily living including activity levels, sleep quality, or symptom self-management can facilitate better monitoring of health and wellness and potentially lead to effective symptom management at an individual level, and contribute to disease surveillance at a population level. Examples include activity tracker data that can inform surveillance of social distancing patterns, and home spirometer and pulse oximetry data that can generate a trajectory of symptom progression in various communities. Finally, in times of “social distancing”, vulnerable populations such as older adults living alone are at greater risk of increased social isolation, which is often referred to as a silent epidemic and great health risk [18]. Digital tools have the potential to connect individuals for the delivery of social services, and creation of virtual peer support groups and connected communities including friends and family members. This current pandemic has highlighted the need for accessible and secure tools that may include video-conferencing, synchronous and asynchronous communication, and even more sophisticated features such as virtual reality and augmented reality, designed for audiences with diverse abilities with the goal to promote social connectedness in times of physical distancing.

### Smart devices and sensors

Smartphones and other wearable smart devices contain research-grade sensors that are capable of shedding light on at least a subset of COVID-19 symptoms which include fever, fatigue, dry-cough, and shortness of breath. For example, the temperature recorded by fingerprint sensors, which are now standard on most modern smartphones, has previously been used to successfully predict fever [19]. In addition, activity sensors such as the accelerometer have been used to detect fatigue. While high resolution computed tomography (CT) images of a patient’s lung may provide a more reliable indicator of infection, the high cost and low scalability make this approach infeasible to apply widely at the general population level. On the other hand, smartphones are currently pervasive with high penetrance even in low and middle-income countries and their high-quality sensor data can be used at next to no cost to measure a subset of important COVID-19 symptoms as a screening tool to identify individuals that may require more extensive evaluation or testing.

## Public health informatics

We present here six considerations of the role of public health informatics in the COVID-19 pandemic. These represent a broad range of topics, from information systems for the monitoring and dissemination of accurate information to the public, to leveraging existing evidence currently available in a huge corpus of virus infection- and pandemic-related research, to building more realistic models of disease risk, spread, and effect of societal interventions, to as-yet poorly understood post-pandemic effects on public health.

### Information systems for COVID-19 monitoring

A critical need for any strategy that addresses COVID-19 is adequate disease monitoring. At the level of cases and deaths, several efforts around the world have arisen to maintain and display official counts, including by researchers at Johns Hopkins University (https://coronavirus.jhu.edu/map.html) and reporters at the New York Times (https://www.nytimes.com/interactive/2020/world/coronavirus-maps.html). These and other efforts rely on reports obtained from heterogeneous sources, many of which capture and store data differently, requiring that informaticians process and display data effectively. Case and death counts are helpful and widely used by healthcare systems, policy makers, governmental institutions, and the general public. However, they are notoriously biased given the differing availability and use of lab-based tests to determine COVID-19 case status at various locations.

More comprehensive efforts to track the true impact of COVID-19 necessitate appropriate wide-scale testing of SARS-CoV-2. Knowledge of who carries the virus regardless of symptom or disease status enables efficient prevention of further transmission, the proper identification of risk factors that lead to divergent symptoms, and adequate preparation of healthcare systems to treat patients who are carriers while minimizing risk to providers and patients who have not been infected. Design and deployment of population-level testing should be a primary goal for the effective containment of COVID-19. In conjunction with apps developed by informaticists, contact tracing along with case isolation can proceed effectively to control outbreaks [8]. Such efforts are thought to have curtailed the spread of COVID-19 in Singapore and South Korea. Because it is unlikely in countries like the U.S., that the federal or local governments, or many citizens would use contact tracing without ensuring individual-level data is safeguarded, various informaticists are engaged in efforts to create privacy-preserving contact tracing apps.

SARS-CoV-2 containment was not successful in most countries, due in part to lack of appropriate wide-scale testing which contributed to its undetected transmission. Ultimately, nothing can replace appropriate lab-based viral testing to understand disease transmission, but informatics solutions are helpful to partly overcome testing inadequacies. In the U.S., Canada, and Mexico, COVID Near You (https://covidnearyou.org/) is a citizen participation platform via which any person can contribute their current health status as it relates to COVID-19 symptoms and test results. Aggregation of this individual-level data is being used to track population-level health in real-time. Other data that can be used to fill monitoring gaps includes search engine data (e.g., Google queries for COVID-19-related terms), and to a lesser extent, social media data (e.g., Twitter posts related to COVID-19). Informaticists are leading and contributing to such efforts around the world.

As results of SARS-CoV-2 tests, along with serological assays to detect its seroconversion, become more widely available, retrospective studies can proceed to more accurately determine how COVID-19 spreads and how many true cases existed prior to widespread testing. Informaticians can participate in these efforts that require accounting for test characteristics (sensitivity/specificity) and comparing the characteristics of patients who were actually tested versus those of the underlying population. Ongoing retrospective analyses such as these are critical to gain knowledge necessary to avoid future resurgences of COVID-19.

### Systems for disseminating accurate information related to COVID-19 to the public

An emerging issue that concerns the prevention of COVID-19 is the widespread dissemination of speculation, rumors, half-truths, disinformation, and conspiracy theories by means of popular social media platforms. In order for policies, guidelines, and mandates, that may be updated on a weekly or even daily basis, to reach and be adopted by the general public it is important for relevant, vetted information sources to be clearly identified and potentially pointed to in response to misleading posts. In recent years there have been many exciting efforts to combine natural language processing (NLP), machine learning, and social media scraping to monitor clinical outcomes of interest such as foodborne illnesses [20]. There may be an opportunity to work towards adapting such informatics approaches to monitor and perhaps even combat the dissemination of ‘bad’ information through automated responses that redirect individuals to sources identified as reliable within the scientific community. Rule-based systems such as ‘expert systems’ could be combined with NLP technologies to construct such monitoring and response frameworks. Equally important is the consumer health informatics task of developing clear, concise, and easily navigable informational resources for COVID-19, that summarize up-to-date information and guidelines but also link summary information back to relevant primary sources, attempt to quantify the certainty/reliability of available information, and offer explanations of reasoning whenever such information or guidelines need to be updated.

### Data visualization and analysis systems for rapid assessment of COVID-19 spread

The spread of infectious diseases such as COVID-19 provides a unique opportunity to assess the regional spread and progression of disease at a population level. Differences in pathogenic mechanisms of different diseases responsible for past pandemics imply that the spread of COVID-19 may not be completely predictable based on the observing historical rates of disease transmission. Data on the cumulative number of COVID-19 cases is available at country/regional/city levels and by studying the progression and spread of disease in regions affected close to the time of the initial outbreak, meaningful projections of infection rates can be made for areas which will be affected later. For example, by modeling daily regional cumulative COVID-19 cases, regional differences in the trends can illuminate the comparative effectiveness of different policy decisions and can identify countries and policies that have succeeded in slowing the rate of COVID-19 spread, providing evidence for the adoption of effective public health policies by areas still in the early phases of the pandemic. Presenting this information to the public using data visualization methods in an important informatics activity.

### Synthesizing evidence to understand COVID-19 origins, spread, and prevention

As of April 11, 2020, there are more than 3700 manuscripts published or posted at PubMed, BioRxiv, and MedRxiv on COVID-19 from researchers all over the world (https://www.ncbi.nlm.nih.gov/research/coronavirus/). These manuscripts cover a wide spectrum of important topics that can help us to understand the critical aspects of clinical and public health impacts of COVID-19, including the disease mechanism, diagnosis, treatment, prevention, viral infection, replication, pathogenesis, transmission, viral host-range, and virulence. On the other hand, the amount of information is increasingly overwhelming for stakeholders, policymakers, researchers and interested parties to comprehend. A systematic review, which is a type of literature review that uses systematic methods to collect secondary data and critically appraise research studies, can be useful in synthesizing the existing evidence of COVID-19 related research findings. In particular, meta-analysis plays a central role in the systematic review in quantitatively synthesizing evidence from multiple scientific studies which address related questions.

Manual literature review is time consuming and, more importantly, it is challenging to keep up-to-date with the rapidly increasing volume of literature. Medical informatics tools can improve the efficiency and scalability of up-to-date evidence synthesis for COVID-19 related research. For example, clinical natural language processing (NLP) tools can be used for literature screening and information retrieval. Software such as Abstractr [21–23] and DistillerSR (https://www.evidencepartners.com/) has been used to reduce manual effort in literature screening. Beyond literature screening, DistillerSR is also a useful tool for the management of the multi-step workflow of systematic review process. Recently, DistillerSR made its tool freely available for systematic reviewers and researchers to conduct systematic reviews related to COVID-19. For meta-analysis, tools such as Comprehensive Meta-Analysis (CMA) (https://www.meta-analysis.com/), RevMan (https://training.cochrane.org/online-learning/core-software-cochrane-reviews/revman), and macros in Stata (https://www.stata.com/), are available for standard meta-analyses. However, for COVID-19 related research, more sophisticated methods are needed in order to address unique features related to this topic. For example, the quality of the reported findings in the above-mentioned 3700 manuscripts is expected to be highly heterogeneous, especially for those manuscripts that have not been peer-reviewed. It is critically important to properly account for such heterogeneity across studies. Furthermore, the reported findings may be subject to more severe publication bias and outcome reporting bias [24], as the analysis of the data and reporting of the analysis results are likely to be based on different protocols. Visualization tools, sensitivity analyses, and inference based on bias correction models can be useful in evaluating the quality of the evidence [25–31]. In addition, novel visualization tools, such as the tornado plot in a cumulative meta-analysis [32], will be valuable for presenting how the cumulative evidence on answering a COVID-19 related question evolves over time. R packages including ‘meta’, ‘metafor’, ‘metasens’, ‘netmeta’, ‘mvmeta’, mada’ and ‘xmeta’ are useful for advanced meta-analyses with these needs. Finally, online platforms for meta-analysis, such as programs with shiny interfaces, are in great need for offering convenience to COVID-19 researchers in summarizing and synthesizing results.

### Advanced, more realistic models of disease spread to guide policymaking

Differential equation-based epidemiological models such as the Susceptible-Infected-Recovered (SIR) or Susceptible-Exposed-Infected-Recovered (SEIR) models and their variants are key workhorses for studying infectious disease dynamics. These models have been widely used in making projections and informing policy-makers in constructing mitigation strategies for the disease. One weakness of these models is that they treat individuals in a given population as homogeneous, with constant risk rates, exposure rates, infection rates, and recovery/death rates throughout the larger group. This is a gross oversimplification which is a primary factor of the models’ limited predictive accuracy. Statisticians have been engaging in COVID-19 efforts with statistical models using functional data or time series modeling techniques. These models often use covariates or latent factors to account for population heterogeneity and provide uncertainty quantification, thus improving on a weakness of the SEIR models. However, these models do not present the dynamic infectious disease process which may limit their interpretability and accuracy in forecasting. One key area of quantitative research that can emerge from this COVID-19 crisis is hybrid epidemiology-statistical models. That is, models based on SIR or SEIR frameworks that stochastically show the transition probabilities as differing according to person or environmental covariates, accounting for clustering effects, and effectively propagating uncertainty in the forecasting. These can combine the strengths of each type of model, and given the broad availability of large scale data on mobility, density, demographics, etc. that vary in different communities, they can produce much more realistic models and more accurate projections to guide policymaking.

### Secondary effects of COVID-19 on public health and well-being

The COVID-19 pandemic has resulted in unprecedented disruption to the healthcare system. In addition to understanding the direct health impacts of the disease, there is a public health need to understand the secondary effects of COVID-19-related healthcare disruption on access to and timeliness of care for other urgent conditions, and resultant effects on health outcomes. Prioritizing healthcare resources for COVID-19 patients and efforts to depopulate healthcare settings in order to reduce healthcare-related disease transmission has resulted in reduced access to care for patients across the spectrum of clinical need and severity including delayed access to surgery for cancer patients, organ transplant recipients, and others with time-sensitive conditions. Public health informatics can play an important role in informing our understanding of how the effects of healthcare disruption propagate across a community, affecting access to care, and population health. Answering questions about the effect of healthcare disruption on population health requires three components: (1) access to data on healthcare utilization and outcomes, (2) data on timing and types of public health and hospital-level interventions, and (3) causal inference methodologies that support our ability to draw conclusions about the causal effects of these interventions. Data on health care utilization and outcomes can be obtained from a variety of sources including individual and multi-institutional EHR data and claims databases. Data on public health interventions are already being compiled by researchers, including national and international databases of policy changes (https://is.gd/CQs6th, https://is.gd/LvvUiz, https://is.gd/mlCu2I). Finally, disentangling the causal impacts of COVID-19 itself; interventions at the local, state, and federal level; and interventions and innovation at the individual health system level requires the rigorous implementation of study designs and analytic methods for causal inference. A number of techniques in common use in health services and econometrics research can be harnessed for this purpose including interrupted time series and difference-in-difference designs [33].

### Real-time monitoring via social media

The total number of users of social media continues to grow worldwide, resulting in the generation of vast amounts of data. Popular social networking sites such as Facebook, Twitter, and Instagram dominate this sphere. About 500 million tweets and 4.3 billion Facebook messages are posted every day (https://www.gwava.com/blog/internet-data-created-daily). A Pew Research Report (http://www.pewinternet.org/fact-sheet/social-media/) states that nearly half of adults worldwide and two-thirds of all American adults (65%) use social networking. The report states that of the total users, 26% have discussed health information, and, of those, 30% changed behavior based on this information and 42% discussed current medical conditions. Advances in automated data processing, machine learning, and NLP present the possibility of utilizing this massive data source for biomedical and public health applications, if researchers address the methodological challenges unique to this media. When events such as the COVID-19 pandemic sweep the world, the public turns to social media. While there is a general belief that most of the content is not useful, adequate collection, filtering, and analysis could reveal potentially useful information for assessing public sentiment. Furthermore, given the delay and shortage of available testing in the United States, social media could provide a near real-time monitoring capability (e.g. the Penn COVID-19 U.S. Twitter map, https://is.gd/L58ggA), giving insights into the true burden of disease. Preliminary work in this direction is under review. The archived version of the paper, with a training dataset and annotation guidelines as supplementary material, is available [34].

Although social media text mining research for health applications is still incipient, the domain has seen a surge in interest in recent years. Numerous studies have been published of late in this realm, including studies on pharmacovigilance [35], identifying user behavioral patterns [36], identifying user social circles with common experiences (like drug abuse) [37], monitoring malpractice [38], and tracking infectious/viral disease spread [39, 40]. Population and public health topics are most addressed, although different social networks may be suitable for specific targeted tasks. For example, while Twitter data has been utilized for surveillance and content analysis, a significant portion of research using Facebook has focused on communication rather than lexical content processing [41, 42]. For health monitoring and surveillance research from social media, the most common topic has been influenza surveillance [43, 44]. From the perspective of informatics and NLP, proposed techniques have typically been in the areas of data collection (e.g., keywords and queries) [45, 46], text classification [47, 48], and information extraction [49]. While innovative approaches have been proposed, there is still a lot of progress to be made in this domain.

Effective utilization of the health-related knowledge contained in social media will require a joint effort by the research community, and bringing together researchers from distinct fields including NLP, machine learning, data science, biomedical informatics, medicine, pharmacology, and public health. The knowledge gaps among researchers in these communities need to be reduced by community sharing of data and the development of novel applied systems.

## Summary

The COVID-19 pandemic presents a myriad of challenges and opportunities for research across virtually every scientific discipline, and biomedical informatics is no exception. From the molecular and genetic sciences to population health, researchers in the five domains of biomedical informatics stand to make substantial contributions to addressing these challenges. We hope, through the numerous examples of research we have considered in this editorial, informatics researchers and practitioners can see possible avenues for their work. There is no dearth of opportunities related to COVID-19 for those working in informatics, and it is our hope that informaticians will vigorously explore these as they arise. Furthermore, we hope that those who are not informaticians will appreciate the contributions that informatics researchers can bring to their respective fields as we all seek to address the COVID-19 pandemic and its effects around the world.

## Data Availability

Not applicable.
